# Patterns of pseudoprogression across different cancer entities treated with immune checkpoint inhibitors

**DOI:** 10.1186/s40644-023-00580-9

**Published:** 2023-06-08

**Authors:** Sebastian Mönch, Maurice M. Heimer, Michael Winkelmann, Anne Guertler, Max Schlaak, Amanda Tufman, Najib Ben Khaled, Enrico de Toni, Christoph B. Westphalen, Michael von Bergwelt-Baildon, Julien Dinkel, Philipp M. Kazmierczak, Michael Ingrisch, Nabeel Mansour, Marcus Unterrainer, Lucie Heinzerling, Jens Ricke, Wolfgang G. Kunz

**Affiliations:** 1 Department of Radiology, University Hospital, LMU Munich, Marchioninistr. 15, Munich, 81377 Germany; 2 Department of Dermatology and Allergy, University Hospital, LMU Munich, Munich, Germany; 3 Department of Medicine V, University Hospital, LMU Munich, Munich, Germany; 4 Department of Medicine II, University Hospital, LMU Munich, Munich, Germany; 5 Department of Medicine III, University Hospital, LMU Munich, Munich, Germany; 6 Comprehensive Cancer Center München-LMU (CCCM LMU ), LMU Munich, Munich, Germany; 7 Clinical Data Science, LMU Munich, Munich, Germany; 8 Department of Dermatology, Venerology and Allergology, Charité – University hospital Berlin, Berlin, Germany

**Keywords:** Immune Checkpoint inhibitors, Pseudoprogression, Cancer, Atypical response patterns, Immune related adverse events

## Abstract

**Background:**

Pseudoprogression (PsPD) is a rare response pattern to immune checkpoint inhibitor (ICI) therapy in oncology. This study aims to reveal imaging features of PsPD, and their association to other relevant findings.

**Methods:**

Patients with PsPD who had at least three consecutive cross-sectional imaging studies at our comprehensive cancer center were retrospectively analyzed. Treatment response was assessed according to immune Response Evaluation Criteria in Solid Tumors (iRECIST). PsPD was defined as the occurrence of immune unconfirmed progressive disease (iUPD) without follow-up confirmation. Target lesions (TL), non-target lesions (NTL), new lesions (NL) were analyzed over time. Tumor markers and immune-related adverse events (irAE) were correlated.

**Results:**

Thirty-two patients were included (mean age: 66.7 ± 13.6 years, 21.9% female) with mean baseline STL of 69.7 mm ± 55.6 mm. PsPD was observed in twenty-six patients (81.3%) at FU1, and no cases occurred after FU4. Patients with iUPD exhibited the following: TL increase in twelve patients, (37.5%), NTL increase in seven patients (21.9%), NL appearance in six patients (18.8%), and combinations thereof in four patients (12.5%). The mean and maximum increase for first iUPD in sum of TL was 19.8 and 96.8 mm (+ 700.8%). The mean and maximum decrease in sum of TL between iUPD and consecutive follow-up was − 19.1 mm and − 114.8 mm (-60.9%) respectively. The mean and maximum sum of new TL at first iUPD timepoint were 7.6 and 82.0 mm respectively. In two patients (10.5%), tumor-specific serologic markers were elevated at first iUPD, while the rest were stable or decreased among the other PsPD cases (89.5%). In fourteen patients (43.8%), irAE were observed.

**Conclusions:**

PsPD occurred most frequently at FU1 after initiation of ICI treatment. The two most prevalent reasons for PsPD were TL und NTL progression, with an increase in TL diameter commonly below + 100%. In few cases, PsPD was observed even if tumor markers were rising compared to baseline. Our findings also suggest a correlation between PsPD and irAE. These findings may guide decision-making of ICI continuation in suspected PsPD.

**Supplementary Information:**

The online version contains supplementary material available at 10.1186/s40644-023-00580-9.

## Background

The introduction of immunotherapies and most prominently immune checkpoint inhibitors (ICI) to the clinical armamentarium has improved clinical outcomes in oncology [1]. ICI are immunoregulatory monoclonal antibodies designed to target inhibitory checkpoints of the immune system, such as cytotoxic T-lymphocyte-associated protein 4 (CTLA-4), programmed death-ligand 1 (PD-L1), or programmed death-1 (PD-1). The antitumor response is thereby stimulated [2]. Originally, clinical efficacy was demonstrated in melanoma patients. In recent years, a substantial clinical benefit was shown for ICI in a broad spectrum of other solid tumors which has led to a surge in oncological indications [3, 4].

Response assessment to ICI is complicated by atypical response patterns, most notably pseudoprogression (PsPD), which is associated with favorable long-term survival as seen in conventional response patterns, but also hyperprogression [5, 6]. To account for atypical response patterns like PsPD, the immune Response Evaluation Criteria in Solid Tumors (iRECIST) criteria were developed for cross-sectional imaging [7]. The category unconfirmed progressive disease (iUPD) was introduced, to recommend short-term follow-up within 4–8 weeks to help differentiate PsPD from true progression [7]. PsPD is a category defined as transient increase in tumor size or occurrence of new tumor lesions with response seen with ICI continuation at later follow-up [3, 8].

Initially, PsPD was observed in metastatic melanoma [9], yet it is also seen in other solid tumors treated with ICI [10]. The incidence of PsPD amounts to 2–10%, depending on tumor entity and PsPD definition [9, 11]. Although PsPD appears to occur less frequently in non-melanoma solid tumors [4, 12], robust data remain scarce [13, 14]. The timely differentiation of PsPD and true progressive disease is highly desirable to help guide the management of oncological therapy. However, currently there are no imaging criteria to improve differentiation of iUPD to identify PsPD.

In this study, we characterized PsPD cases comprehensively. This includes the response assessment according to iRECIST, organ distribution, quantification of absolute and relative tumor burden changes, the association with serological tumor markers, and correlation to irAE.

## Methods

### Study population

Patients with metastatic solid tumors who were treated with ICI at the Comprehensive Cancer Center Munich-Ludwig-Maximilian University Munich (CCCM^LMU^) were included in this study; treatment strategies included single-agent ICI, double-agent ICI, combined ICI and chemotherapy, or targeted therapy. To identify patients with PsPD, we screened ICI-treated cohorts for the tumor entities melanoma, lung cancer, sarcoma, genitourinary cancer, cancer of unknown primary, ENT cancer, mesothelioma, and gastrointestinal tumors including colorectal carcinoma and hepatocellular carcinoma, for which a prospective registry was available.

Patients were identified through review of medical healthcare records and available imaging reports. We were not able to assess the true rate of PsPD in all solid tumor entities as there are no ubiquitous prospective registries and all patients were treated as part of routine clinical care. Medical records and imaging studies were retrospectively reviewed with the approval of the LMU Munich Institutional Review Board (Ethikkommission der Medizinischen Fakultät der Ludwig-Maximilians-Universität München) with the waiver for informed consent.

Inclusion Criteria:

1. Patients with metastatic solid tumors treated with ICI, either alone or in combination treatment strategies.
2. Available cross-sectional CT, MR or PET imaging studies at baseline (≤ 4 weeks before ICI initiation) and at least two follow-up timepoints (FU1 and FU2), with FU 8–12 weeks after the baseline examination. FU2 was 4–8 weeks after FU1.
3. Occurrence of PsPD defined as immune unconfirmed progressive disease (iUPD) without follow-up confirmation as per iRECIST [7].

Exclusion criteria:

1. Any change in initial oncological treatment protocol after iUPD assessment.
2. Any ICI discontinuation before follow-up timepoint 2 (FU2).
3. Isolated imaging findings that are well-established mimickers of progression such as mediastinal and hilar lymphadenopathy and sarcoid-like pulmonary lesions (occurring in immune-related adverse events [15]) were not considered for definition of iUPD.

ICIs were given as recommended by the providers. A detailed list of treatment medications for all PsPD patients is provided in the appendix (Supplementary Table S1).

By patterns of pseudoprogression we describe various characteristics of patients with pseudoprogression such as reason of pseudoprogression (progression due to target lesions, non-target lesions, new target lesions, new non-target lesions, or multiple combinations of these reasons), time point of pseudoprogression after initiation of ICI therapy, or occurrence of irAE.“.

### Imaging response assessment

Therapy response was assessed in accordance to iRECIST [7]. PsPD was defined as any occurrence of immune unconfirmed progressive disease (iUPD) without follow-up confirmation. Target lesions (TL), non-target lesions (NTL), new lesions (NL, either as target or non-target NL; new TL, new NTL) were individually segmented and annotated. TL and new TL were evaluated as a separate sum of diameters (SOD). Timepoint of iUPD was documented to calculate time to PsPD from baseline. PsPD was then categorized as early (within the first 12 weeks of therapy) and late PsPD (after 12 weeks of therapy) [3]. We aligned our efficacy reporting standards with the Trial Reporting in Immuno-Oncology (TRIO) consensus statement by the American Society of Clinical Oncology (ASCO) and the Society of Immunotherapy of Cancer (SITC) [16].

All imaging analyses were performed with dedicated trial reporting software mintLesion 3.8 (mint Medical GmbH; Heidelberg, Germany). Organ distribution for TL, NTL, new TL, and new NTL was documented and sub-grouped as nodal lesions, visceral lesions, non-visceral lesions, and a combination of these options as previously described [17].

### Serological tumor marker assessment

Tumor-specific serological markers were collected closest to all imaging timepoints if available. The course was classified as concordant if PsPD coincided with decrease in markers, as discordant if markers increased, and as stable if the markers remained unchanged. The following serological tumor makers were analyzed according to tumor entity: S100 protein, Cytokeratin-Fragment 21 − 1 (CYFRA 21 − 1), Carcinoembryonic antigen (CEA), alpha feto protein (AFP), Cell Carcinoma Antigen (SCC), Cancer Antigen 19 − 9 (CA 19 − 9), Cancer Antigen 125, (CA 125), neuron specific enolase (NSE). Additionally, lactate dehydrogenase (LDH) as a non-specific serological tumor marker was analyzed.

### Toxicity assessment

Immune-related adverse events (irAE) were reported in line with the consensus reporting guidelines on efficacy and toxicity (JCO TRIO) [16] and graded along the Common Terminology Criteria for Adverse Events (CTCAE) v5.0. The timepoint of first clinical presentation of irAE was compared with the timepoint of PsPD.

### Statistical analysis

Descriptive statistics were conducted to obtain distribution of tumor entity, ICI administration, tumor response rates, temporal occurrence of PsPD, reasons for PsPD, tumor dynamics around PsPD, course of serological tumor markers, and occurrence of irAEs. Differences in PsPD parameters between patients with melanoma and patients with non-melanoma tumor entities as well localization of the tumor were analyzed with group comparisons using the χ2-test, Mann-Whitney U test, unpaired students T test, analysis of variance, or Kruskal-Wallis test depending on the type of variables analyzed. Data are generally displayed as N (%) or mean, if not indicated otherwise. Statistical significance was assumed at P < 0.05. The statistical analysis was performed with the Statistical Package for Social Sciences Version 28.0.1.1. (SPSS, Chicago, IL, USA) and with Excel 2022 software (Microsoft, Redmond, WA, USA).

## Results

### Patient characteristics

**Fig. 1:**
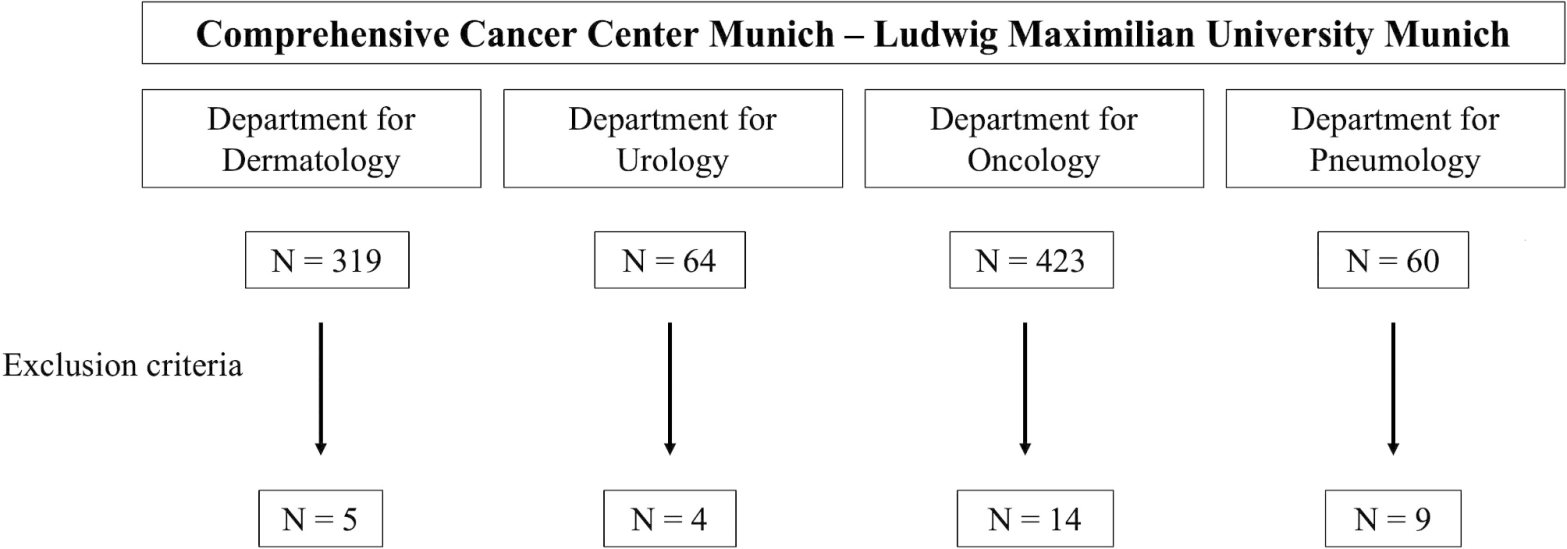
Flow diagram patient selection

Thirty-two patients with different solid tumors who received single-agent ICI, double-agent ICI, combined ICI and chemotherapy, or target therapy were included (Fig. 1). The mean age of this study population was 66.7 ± 13.6 years with a proportion of 21.9% females. The three most common entities were non-small cell lung cancer (NSCLC) with 21.9%, hepatocellular carcinoma (HCC) with 18.8%, and melanoma with 15.6% (Additional file 1 ). The most frequent ICI treatments were monotherapies with Pembrolizumab (34.4%) or Nivolumab (25.0%), followed by double-agent ICI consisting of Ipilimumab in combination with Nivolumab (15.6%) or Durvalumab in combination with Tremelimumab (9.4%) (Additional file 2 ). Additional information on previous therapies is provided in Additional file 3 .

### Temporal occurrence and reason for pseudoprogression

**Fig. 2:**
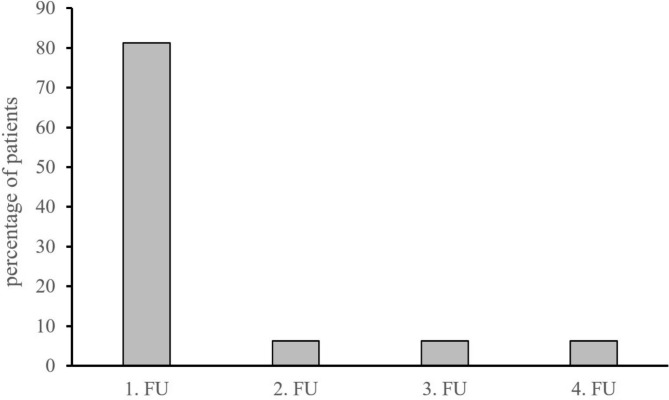
Temporal occurrence of pseudoprogression. FU follow-up examination

Twenty-six patients (81.3%) had first unconfirmed progressive disease (iUPD) at follow-up examination 1 (FU1, Fig. 2). Less often it occurred at the second (FU2), third (FU3), or even as late as the fourth follow-up exam (FU4) (each 6.3%). No PsPD was observed past follow-up examination 4.

**Fig. 3:**
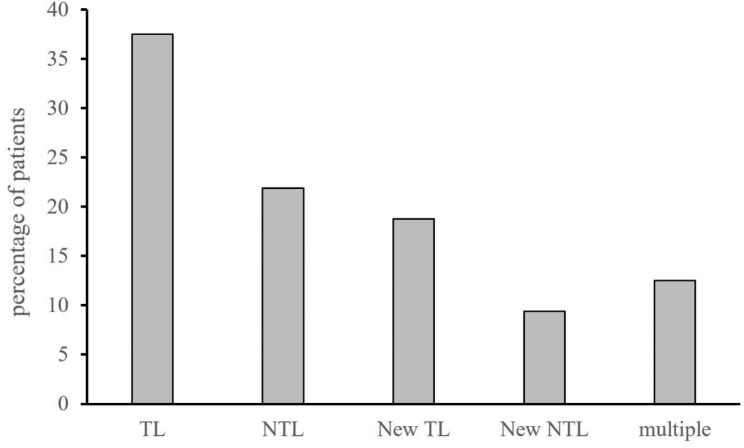
Reason for pseudoprogression. TL target lesion, NTL non-target lesion, New TL new target lesion, New NTL new non-target lesion, multiple combination of reasons

Overall, PsPD was based on target lesion progression in twelve patients (37.5%), on non-target lesion progression in seven patients (21.9%), on new lesion appearance in six patients (18.8%), on new non-target lesion occurrence in three (9.4%), and on multiple levels in four patients (12.5%) (Fig. 3).

### Tumor dynamics around pseudoprogression

**Fig. 4:**
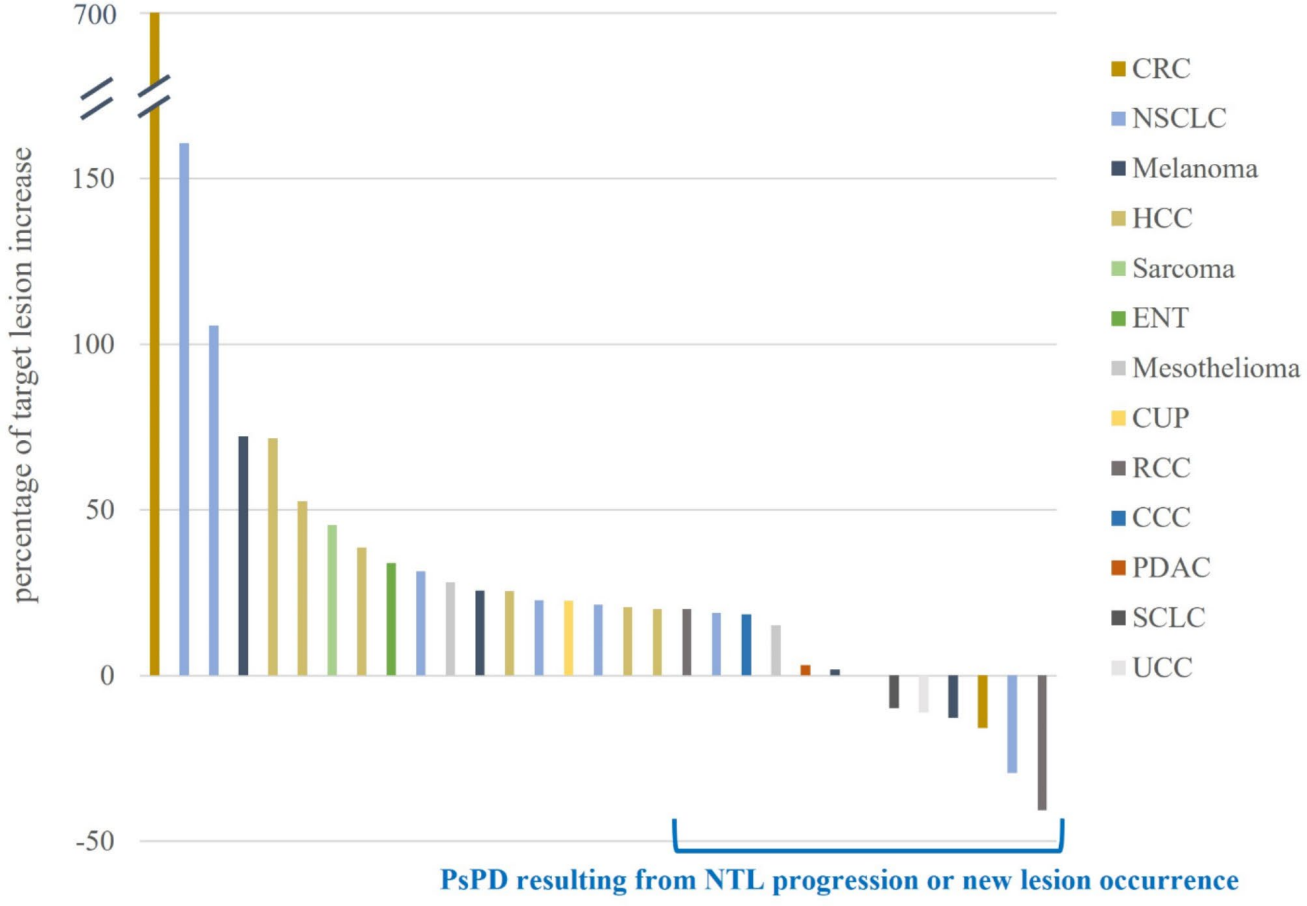
Change of tumor size from baseline to pseudoprogression. NSCLC Non-small cell lung cancer, RCC renal cell carcinoma, HCC hepatocellular carcinoma, ENT ear nose throat tumor, CUP carcinoma of unknown primary, CRC colorectal carcinoma, CCC cholangiocellular carcinoma, PDAC pancreatic ductal adenocarcinoma, SLCL small cell lung cancer, UCC urothelium cell carcinoma. NTL non-target lesion

**Fig. 5:**
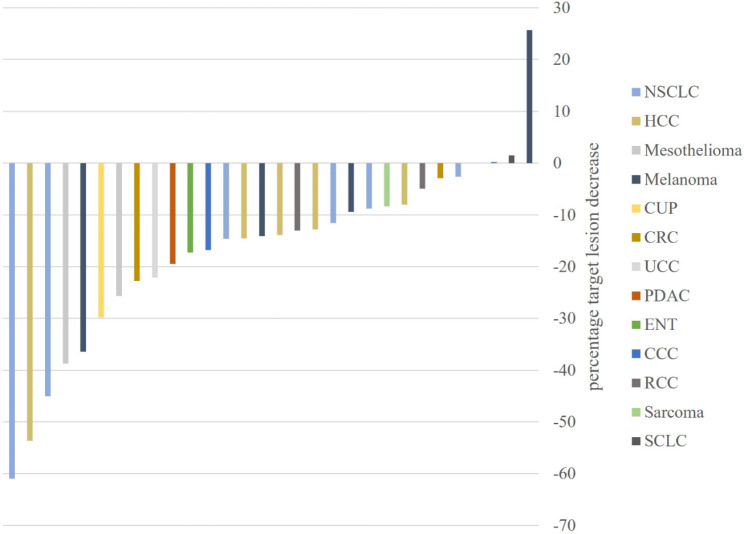
Tumor response following pseudoprogression. NSCLC Non-small cell lung cancer, RCC renal cell carcinoma, HCC hepatocellular carcinoma, ENT ear nose throat carcinoma, CUP carcinoma of unknown primary, CRC colorectal carcinoma, CCC cholangiocellular carcinoma, PDAC pancreatic ductal adenocarcinoma, SLCL small cell lung cancer, UCC urothelium cell carcinoma

The mean target sum of all patients at baseline imaging was 69.7 mm ± 55.6 mm. The maximum increase in sum of TL was 96.8 mm (+ 700.8%) (Fig. 4). The mean increase in sum of TL was 19.8 mm ± 25.5 mm (47%) and the median increase in sum of TL was 13.0 mm. The mean and maximum decrease in sum of TL between the first iUPD timepoint and the next follow-up was − 19.1 mm ± 28.5 mm (-16.7%) and − 114.8 mm (-60.9%) (Fig. 5). The mean and maximum sum of new TL at first iUPD timepoint was 7.6 mm ± 16.1 and 82.0 mm.

### Course of serological tumor markers at pseudoprogression

**Fig. 6:**
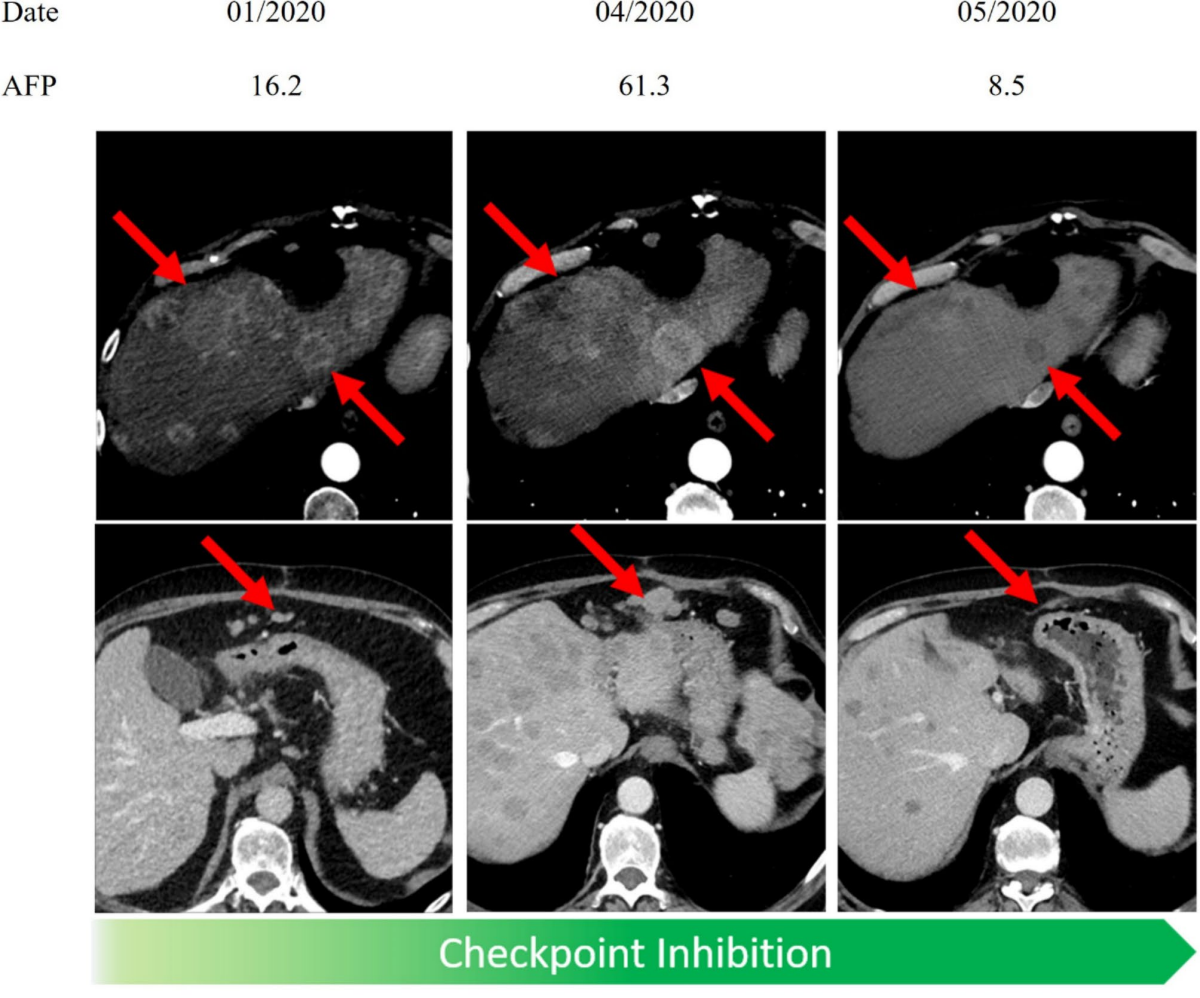
Pseudoprogression in a patient with HCC under Nivolumab and Lenvatinib. Discordant elevation of AFP at PsPD is shown. Red arrows indicate the tumor burden at a specific time point. AFP alpha feto protein, HCC hepatocellular carcinoma

Only a minority of patients with PsPD showed a concordant decrease of entity-specific.

serological tumor markers (10.5%). The majority of patients had stable serologic tumor markers (68.4%). 21.1% showed a discordant increase of tumor markers; as an example one of these patients is demonstrated in Fig. 6. Likewise, only a minority (21.4%) of patients showed elevated LDH levels.

### Occurrence of immune-related adverse events

**Fig. 7:**
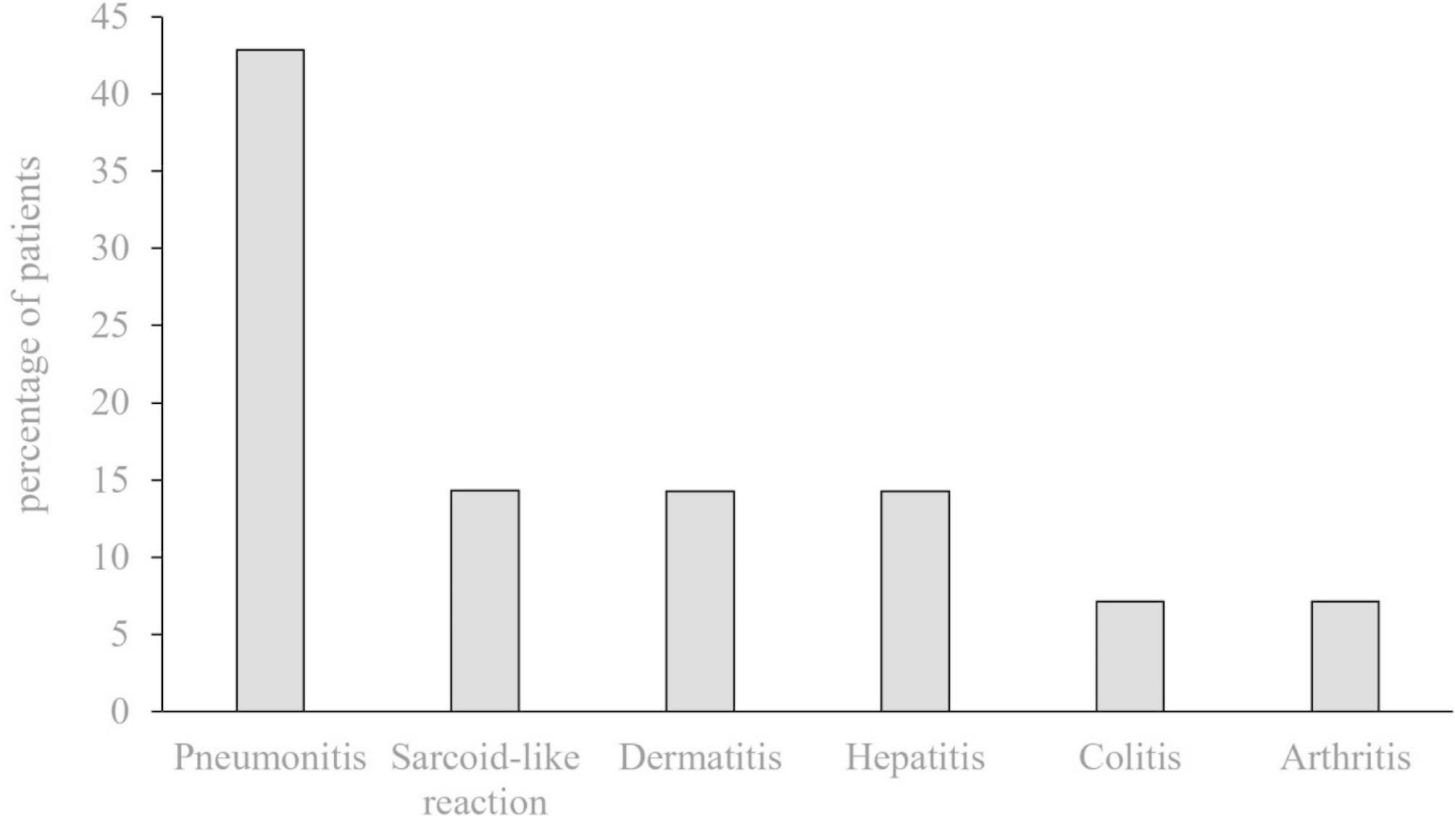
Occurrence of immune-related adverse events. Depicted is the percentage of specific adverse event of those patients with immune-related adverse events

Immune-related adverse events (irAE) were confirmed in fourteen of the thirty-two patients (43.8%). In ten of these fourteen patients the irAE was coincidentally present at the imaging time point of PsPD, in the remaining four patients the irAE did not occur at the timepoint of PsPD. As depicted in Fig. 7, pneumonitis was the most frequent adverse event (42.9%), followed by sarcoid-like reactions (as an independent feature in addition to the actual PsPD), hepatitis, and dermatitis each with 14.3%. Of these fourteen patients with irAE, six (42.9%) had a CTCAE grade 1, six (42.9%) had a CTCAE grade 2, and two (14.2%) had a CTCAE grade 3. No patients with CTCAE grade 4 and 5 were included. Of the two patients with CTCAE grade 3, one had a rapidly improving hepatitis after glucocorticoid administration, in the other patient the irAE occurred three follow-up examinations after the actual timepoint of PsPD.

No significant differences were observed between patients with melanoma and patients with other solid tumors when comparing the timepoint of PsPD, the maximum increase at PsPD, the decrease of TL following PsPD, the presence of irAE, elevated lactate dehydrogenase (LDH) at PsPD, or concordance of tumor specific markers (Additional file 4 ). Likewise, no significant differences were observed when localization subgroups (visceral, nodal, non-visceral versus a combination of these) were analyzed (Additional file 5 ). Also no significant differences were detected when subgroup analyses were performed regarding lung tumors versus other tumors, ICI monotherapy versus association of ICI or Nivolumab alone or Pembrolizumab alone, respectively, versus other ICI treatments (Additional files 6–9 ).

## Discussion

Although atypical response patterns in ICI treatment are rare, they have significant impact upon patient management in oncology. The etiology of PsPD remains unclear. It is hypothesized that it correlates to a delayed therapy response as compared to chemotherapy or a therapy-associated immune cell infiltration [18, 19]. PsPD occurs most frequently at FU1 with an increase in tumor burden regularly less than + 100%. Our study is in alignment with the CheckMate 066 and 067 trials, showing that PsPD in metastatic melanoma was most frequently at initial follow up (FU1) [20]. In a large meta-analysis of eight trials, PsPD in metastatic melanoma occurred most frequently as a result of new lesions with a considerable number of patients that had target lesion response during time of first PD assessment [17]. Patients with PsPD had significantly fewer new CNS metastases compared to PD [17]. In contrast, our study our study showed that PsPD was associated with significant increase of the target and non-target lesion progression.

Currently, PsPD is defined as target lesion progression or presence of one or more new target lesions according to iRECIST [4]. We observed that the second most common reason for PsPD (23.3%) in our cohort was NTL progression.

While elevated LDH has previously been associated to inferior five-year survival in patients with advanced melanoma receiving a combined Nivolumab and Ipilimumab therapy, LDH levels in patients with PsPD in our cohort were normal in most of the cases [21]. These results are in accordance to a single-center study, showing that increased LDH/S100 ratios were linked to lower rates of PsPD in melanoma patients [22]. This is, however, challenged by a study showing that elevated LDH do not affect event-free survival in a similar patient and treatment setting [23].

In this study, two PsPD patients (11.8%) showed highly elevated tumor entity specific serologic markers and five patients (18.5%) demonstrated elevated LDH at the timepoint of PsPD. Considering our data, the role of serological parameters to identify patients with PsPD remains unclear [24]. Therefore, increased LDH and increased tumor entity-specific serologic markers do not reliably exclude the presence of PsPD.

Since ICI are immunoregulatory drugs known not only to stimulate the antitumor immune response but also to aggravate autoimmune effects, we investigated the association of PsPD and irAEs. Initial clinical trials in malignant melanoma treated with CTLA-4 antibodies reported irAE in about one third of patients who were shown to have clinical response [2]. In our cohort, 40% of PsPD patients had an irAE. The literature reports irAE frequency in melanoma patients under ICI treatment at about 31% [16]. These aggregated data suggest that the coincidence of irAE and iUPD may raise suspicion underlying PsPD. This is interesting as the pathophysiology of PsPD is only partially understood. One hypothesis is the infiltration of immune cells into the tumor as a cause for PsPD. However, the true association between irAE and PsPD remains unknown with ICI discontinuation in patients with severe irAE before PsPD could be confirmed in follow-up assessment.

As demonstrated and discussed elsewhere before, the awareness for atypical response patterns such as PsPD in immunotherapy treatment is highly important for the oncologist to correctly assess the success of the ICI therapy applied to a tumor patient. Nonetheless, PsPD and PD at iUPD can currently only be differentiated by follow-up assessment. With regard to initial efficacy studies it was estimated that RECIST v1.1 criteria, which do not account for atypical response patterns, underestimate the benefit of Pembrolizumab in approximately 15% of patients. Patients with PsPD benefit from ICI continuation beyond initial evidence of radiographic progression. Hence, aborting the ICI treatment prematurely may have negative consequences.

This study had several limitations. Firstly, the study design was retrospective and relied on a limited number of patients recruited at a single center. However, detailed analysis of imaging findings in PsPD patients over a variety of solid tumors remains scarce. Secondly, our study does not capture all patients with PsPD within the indicated time frame. Therefore, the true incidence of PsPD across various tumor entities cannot be deducted from this study. Furthermore, absolute frequency of PsPD in certain tumor entities could be biased by the availability of imaging and chart documentation. Subgroup analyses by individual checkpoint inhibitor were not feasible due to limited sample sizes. Lastly, patients who received chemotherapy in combination with ICI were also included; potential bias regarding PsPD is however unlikely.

## Conclusion

PsPD occurred most often at early follow-up (FU1) in a variety of solid tumors, but could also be observed as late as FU4. The two most frequent reasons noted for PsPD were TL und NTL progression. irAE were shown to occur disproportionately often in patients with PsPD which may suggest a correlation. In few cases PsPD was observed with incidental elevation of tumor markers. Our results may help guide decision-making when PsPD is suspected and warrant further prospective validation.

## Electronic supplementary material

Below is the link to the electronic supplementary material.

Supplementary Material 1

Supplementary Material 2

Supplementary Material 3

Supplementary Material 4

Supplementary Material 5

Supplementary Material 6

Supplementary Material 7

Supplementary Material 8

Supplementary Material 9

Supplementary Material 10

## Data Availability

The datasets used and/or analysed during the current study are available from the corresponding author on reasonable request.

## List of abbreviations

CNS: Central nervous system
CT: Computed tomography
CTCAE: Common Terminology Criteria for Adverse Events
CTLA-4: Cytotoxic T-lymphocyte-associated protein 4
FU: Follow-up examination
HCC: Hepatocellular carcinoma
ICI: Immune checkpoint inhibitor
irAE: Immune-related adverse events
iRECIST: Immune Response Evaluation Criteria in Solid
iUPD: Immune unconfirmed progressive disease
LDH: Lactate dehydrogenase
MR: Magnetic resonance
NL: New lesions
NSCLC: Non-small cell lung cancer
NTL: Non-target lesions
PET: Positron emission tomography
PD: Progressive disease
PD-1: Programmed death-1
PD-L1: Programmed death-ligand 1
PsPD: Pseudoprogression
STL: Sum target lesions
TL: Target lesions
