# Supplementary material for: Patterns of pseudoprogression across different cancer entities treated with immune checkpoint inhibitors
Source: Cancer Imaging. 2023 Jun 8;23:58. doi: 10.1186/s40644-023-00580-9 (PMC10249323; doi:10.1186/s40644-023-00580-9)
Supplement: Supplementary file 2 — Supplementary Material 2 [file 40644_2023_580_MOESM2_ESM.docx]

## Table S1. Patient characteristics

| **Patient ID** | **Age** | **Gender** | **Tumor type** | **Tumor stage** | **Mutations** | **Checkpoint inhibitor** | **Concomitant anti tumor therapy** | **Previous therapies** | **irAE** | **CTCAE grade** | **Steroids for irAE** | **irAE concomitant with PsPD** |
| --- | --- | --- | --- | --- | --- | --- | --- | --- | --- | --- | --- | --- |
|  |  |  |  |  |  |  |  |  |  |  |  |  |
| **1** | 51 | f | HCC | pT3 N0 M0 L0 V0 Pn0 R0 G2 | BAP1 T93A mutation | Nivolumab | Lenvatinib | hemihepatectomy right 09/2019 | no | / | / | / |
| **2** | 65 | m | NSCLC | cT4 cN0 cM1VC | TP53 R142P mutation | Pembrolizumab | none | Carboplatin + Paclitaxel 04/2019 - 06/2019 | pneumonitis | 1 | / | no, after |
| **3** | 51 | f | NSCLC | cT3 cN3 cM1b | MET exon 14 skipping mutation | Durvalumab | none | Cisplatin + Pemetrexed 11/2017 - 02/2018, radiotherapy 02/2018 - 04/2018 | pneumonitis | 2 | yes | yes |
| **4** | 63 | m | HCC | pT2 N1 M1 G2 R1 | NA | Nivolumab | Lenvatinib | partial liver resection 09/2019 | no | / | / | / |
| **5** | 62 | m | CCC | T1 N1 M0 | NA | Durvalumab | Gemcitabin + Cisplatin | none | no | / | / | / |
| **6** | 71 | m | NSCLC | cT2b cN1 cM1b | EGFR negative, ALK negative, ROS-1 negative, BRAF negative | Pembrolizumab | none | Carboplatin + Paclitaxel 02 - 03/2016, radiotherapy + Cisplatin 03 - 06/2016 | no | / | / | / |
| **7** | 77 | f | UCC | pT3 pNx R1 V0 pN0 cM1 | TERT: 5p15.33, chr5:1253820, approximately 9.85x more. FGFR1: 8p11.23, chr8:38271114, approximately 8.2x more. NF2: 22q12.2, chr22:29999946, approximately 20.65x. | Atezolizumab | none | nephroureterectomy 07/2019 | no | / | / | / |
| **8** | 81 | m | NSCLC | cT4 cN3 cM1c | NA | Pembrolizumab | none | tumor resection 04/2020 | no | / | / | / |
| **9** | 77 | m | HCC | T3b N0 M0 | NA | Durvalumab + Tremelimumab | none | radiotherapy 11 - 12/2014, tumor resection 01/2015 | pneumonitis | 1 | yes | yes |
| **10** | 68 | m | Sarcoma | pT2b N0 M0 L0 V0 Pn0 G3 | NA | Durvalumab + Tremelimumab | none | none | no | / | / | / |
| **11** | 50 | m | CRC | pT2 pN1 G2 L1 V0 | RAS/BRAF WT, MLH loss of expression; MSS, TMB, 4mut/MB, Her2 score 1+, FISH negative | Pembrolizumab | none | low anterior rectum resection 01/2010, FOLFIRI 02 - 07/2010, regional relapse tumor resection, partial liver resection 03/2014, regional relapse tumor resection 03/2015, Bevacizumab + Capecitabin 04 - 06/2015, partial liver resection 08/2015, FOLFOX + Panitumumab 06/2016, radiotherapy + Capecitabin 02 - 04/2018, local relapse tumor resection 05/2018 | no | / | / | / |
| **12** | 70 | m | Melanoma | pT0 N2b M1d | BRAF V600E | Ipilimumab | none | neck dissection 10/2010, Dabrafenib + Trametinib 04/2013 - 03/2014 | no | / | / | / |
| **13** | 40 | m | PDAC | St. IVa, uC4 uN2 uM1 G3 | Cytokeratin 7 positive, Ki-67 expression 45 %, MSI (high); Cytokeratin 20, CDX 2, TTF-1, and Synaptophysin negative | Pembrolizumab | none | FOLFIRINOX 11/2018 - 02/2019 | no | / | / | / |
| **14** | 75 | m | Mesothelioma | NA | NA | Nivolumab | none | partial resection of lung, pericardium and pleura 12/2016, radiotherapy 01/2018 | no | / | / | / |
| **15** | 91 | m | Melanoma | Tx N0 M1d | BRAF WT, NRAS Q61 mutation, PDL-1 expression > 2% | Pembrolizumab | none | partial lung resection 08/2016 | sarcoid-like lesions | 1 | no | yes |
| **16** | 83 | m | NSCLC | cT4 cN3 cM1 G3 | PDL1 expression 80% | Pembrolizumab | none | none | no | / | / | / |
| **17** | 77 | m | HCC | St. IV | NA | Durvalumab + Tremelimumab | none | partial liver resection and radio frequency ablation of the liver 09/2014, transarterial chemoembolisation + stereotactic radiotherapy of the liver 05/2016, transarterial chemoembolisation of the liver 09/2016, microwave ablation of the liver 05/2017, brachytherapy of the liver 08/2017 | pneumonitis | 3 | yes | no, after |
| **18** | 44 | m | CRC | rpT4b rpNX L1 V1 Pn1 G3 RX | MSI, KRAS mut, BRAF WT | Pembrolizumab | none | neoadjuvant radiotherapy + 5-Fluorouracil 10/2014, low anterior rectum resection 03/2015, resection of a relapse tumor including partial resection of the sacrum and ileocolic resection 02/2017 | sarcoid-like lesions | 1 | no | yes |
| **19** | 64 | f | NSCLC | AJCC St. IVb | PDL-1 expression 10%, TTF-1 and ALK negative, CK7 expression and fokal positivity for Napsin A, KRAS GV12 mutation; nNGM: no further aberrations. | Pembrolizumab | Cisplatin + Pemetrexed | none | no | / | / | / |
| **20** | 76 | m | NSCLC | T2a N3 M0 UICC IIIb | TTF1 negative, PDL-1 negative, CK 56 at most sinularily weakly positive, p40 negative | Nivolumab | none | Cisplatin + Vinorelbin 03/2017, Carboplatin + Vinorelbin 05/2017 | arthritis | 1 | no | yes |
| **21** | 41 | m | CRC | pT1 pN0 cM1a V0 L0 Pn0 R2 | Loss of expression of MLH1 and PMS-2, CTNNB1 T34A mutation, RHOA Y42C mutation, CCND1 P287H mutation, ERBB3 M60R mutation, SMARCA4 E1435G mutation. | Pembrolizumab | none | partial liver resection and rightsided hemicolectomy 08/2016 | no | / | / | / |
| **22** | 66 | m | ENT | rpT2 cN0 M1 L0 V0 Pn0 R0 | NA | Nivolumab | none | radiotherapy and resection of oro- and hypopharynx tumor 2014, tumor resection and neck dissection 06/2015 | dermatitis | 2 | no | no, after |
| **23** | 89 | m | RCC | cN0 M0 | NA | Nivolumab | none | nephrectomy left 1976, Pazopanib 06/2016 | pneumonitis | 1 | yes | yes |
| **24** | 69 | m | CUP | G1 pN1 L1 Pn1 R0 | NA | Atezolizumab | Gemcitabin + Cisplatin | Carboplatin + Paclitaxel 04 - 05/2019 | no | / | / | / |
| **25** | 73 | m | RCC | pT1b L0 V0 G2 R0 | NA | Nivolumab | none | nephrectomy right 02/2020, Sunitinib 06/2016 - 04/2018 | pneumonitis | 2 | yes | yes |
| **26** | 73 | m | SCLC | cT2 cN2 M1 | Ki-67 expression 70-80%, PDL-1 expression 10% | Pembrolizumab | none | Carboplatin + Etoposid 12/2017 - 06/2019, Paclitaxel 07 - 09/2019 | no | / | / | / |
| **27** | 65 | m | HCC | BCLC C N0 M0 | NA | Nivolumab | Lenvatinib | none | colitis | 2 | yes | no, after |
| **28** | 81 | m | Melanoma | AJCC St. IV, pT4b N3b M1d | BRAF WT | Ipilimumab + Nivolumab | none | none | hepatitis | 2 | yes | yes |
| **29** | 66 | m | HCC | BCLCB/  UICC IIIA, cT3 N0 M0 | NA | Ipilimumab + Nivolumab | none | Selective internal radiation therapy and Brachytherapie 03/2020, Lenvatinib 06/2020 - 04/2021, partial liver resection 06/2021, Suratinib 08 - 09/2021 | no | / | / | / |
| **30** | 40 | f | Melanoma | AJCC St. IV, pT4b N0 M0 | BRAF WT, KIT WT | Ipilimumab + Nivolumab | none | Interferon alpha 01/2015 - 08/2016, partiel lung resektion 11/2017 | no | / | / | / |
| **31** | 63 | f | Mesothelioma | T2 | Calretinin and WT-1 positive, PDL-1 negative | Ipilimumab + Nivolumab | none | Cisplatin + Pemetrexed followed by Pemetrexed + Carboplatin + UAC 5 + Avastin 03 - 12/2017 | dermatitis | 2 | yes | yes |
| **32** | 65 | f | Melanoma | AJCC St. IV | HLA-A*02:01 positiv | Ipilimumab + Nivolumab | none | none | hepatitis | 3 | yes | yes |

f female, m male, NA not available, St. Stadium, / not applicaple, AJCC American Joint Committee on Cancer, UICC International Union Against Cancer, NSCLC Non-small cell lung cancer, RCC renal cell carcinoma, HCC hepatocellular carcinoma, ENT ear nose throat carcinoma, CUP carcinoma of unknown primary, CRC colorectal carcinoma, CCC cholangiocellular carcinoma, PDAC pancreatic ductal adenocarcinoma, SLCL small cell lung cancer, UCC urothelium cell carcinoma.
