# Supplementary material for: Patterns of pseudoprogression across different cancer entities treated with immune checkpoint inhibitors
Source: Cancer Imaging. 2023 Jun 8;23:58. doi: 10.1186/s40644-023-00580-9 (PMC10249323; doi:10.1186/s40644-023-00580-9)
Supplement: Supplementary file 3 — Supplementary Material 3 [file 40644_2023_580_MOESM3_ESM.docx]

**Table S2. Comparison of patients with melanoma versus non-melanoma**

|  | Melanoma  (N = 5) | Non-melanoma  (N = 27) | P value |
| --- | --- | --- | --- |
| PsPD at FU1 | 100.0 % (N = 5) | 77.8 % (N=21) | 0.242 |
| Max. increase of TL (cm) | 2.0 ± 10.5 | 14.9 ± 23.5 | 0.377 |
| Max. decrease of TL (cm) | -15.3 ± 18.9 | -17.8 ± 16.5 | 0.767 |
| Presence of irAE | 60.0 % (N = 3) | 42.3 % (N = 11) | 0.467 |
| Elevated LDH | 20.0 % (N = 1) | 17.4 % (N = 4) | 0.890 |
| concordant tumor specific markers | 0.0 % (N = 0) | 7.4 % (N = 2) | 0.407 |

PsPD pseudoprogression, irAE immune-related adverse event, TL target lesion sum, max. maximum, LDH Lactate dehydrogenase, FU follow-up examination
