# Supplementary material for: Patterns of pseudoprogression across different cancer entities treated with immune checkpoint inhibitors
Source: Cancer Imaging. 2023 Jun 8;23:58. doi: 10.1186/s40644-023-00580-9 (PMC10249323; doi:10.1186/s40644-023-00580-9)
Supplement: Supplementary file 4 — Supplementary Material 4 [file 40644_2023_580_MOESM4_ESM.docx]

**Table S3. Comparison of patients according to tumor localization**

|  | Visceral  (N = 10) | Nodal  (N = 6) | Non-visceral  (N = 11) | Combined  (N = 5) | P value |
| --- | --- | --- | --- | --- | --- |
| PsPD at FU1 | 90.0 % (N = 9) | 83.3 % (N = 5) | 72.7 % (N = 8) | 80.0 % (N = 4) | 0.789 |
| Max. increase of TL (cm) | 16.8 ± 15.1 | 6.1 ± 5.6 | 1.4 ± 15.1 | 38.7 ± 38.2 | 0.009 |
| Max. decrease of TL (cm) | -22.7 ± 17.6 | -11.3 ± 7.3 | -15.9 ± 14.8 | -17.5 ± 24.6 | 0.654 |
| Presence of irAE | 30.0 % (N = 3) | 50.0 % (N = 3) | 54.5 % (N = 6) | 50.0 % (N = 2) | 0.467 |
| Elevated LDH | 22.2 % (N = 1) | 20.0 % (N = 1) | 11.1 % (N = 1) | 20.0 % (N = 1) | 0.935 |
| Concordant tumor markers | 10.0 % (N = 1) | 16.7 % (N = 1) | 0.0 % (N = 0) | 0.0 % (N = 0) | 0.768 |

PsPD pseudoprogression, irAE immune-related adverse event, TL target lesion sum, max. maximum, LDH lactate dehydrogenase, FU follow-up examination
