# Supplementary figures and images for: Patterns of pseudoprogression across different cancer entities treated with immune checkpoint inhibitors
Source: Cancer Imaging. 2023 Jun 8;23:58. doi: 10.1186/s40644-023-00580-9 (PMC10249323; doi:10.1186/s40644-023-00580-9)

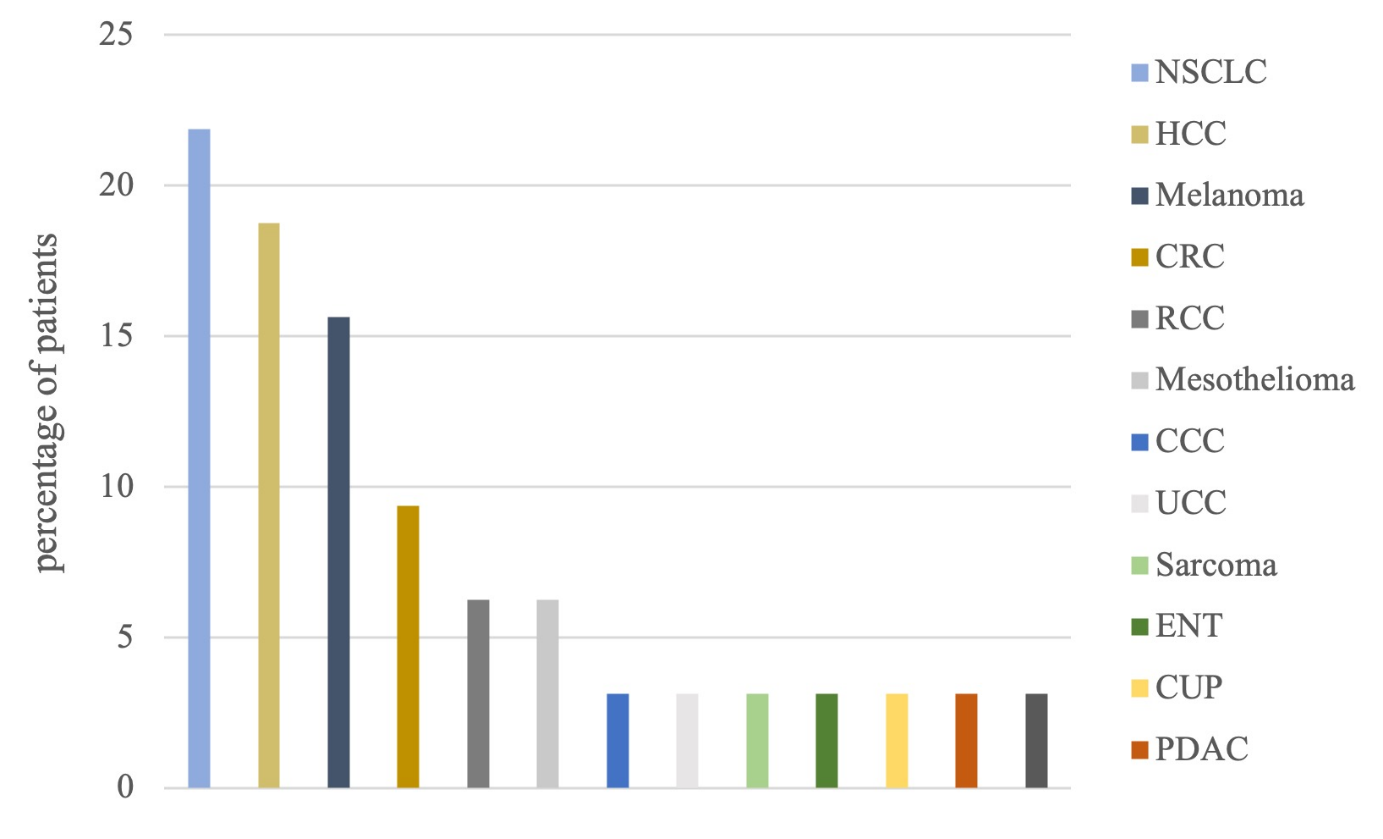

Supplement: Supplementary file 5 — Supplementary Material 5 [file 40644_2023_580_MOESM5_ESM.docx]

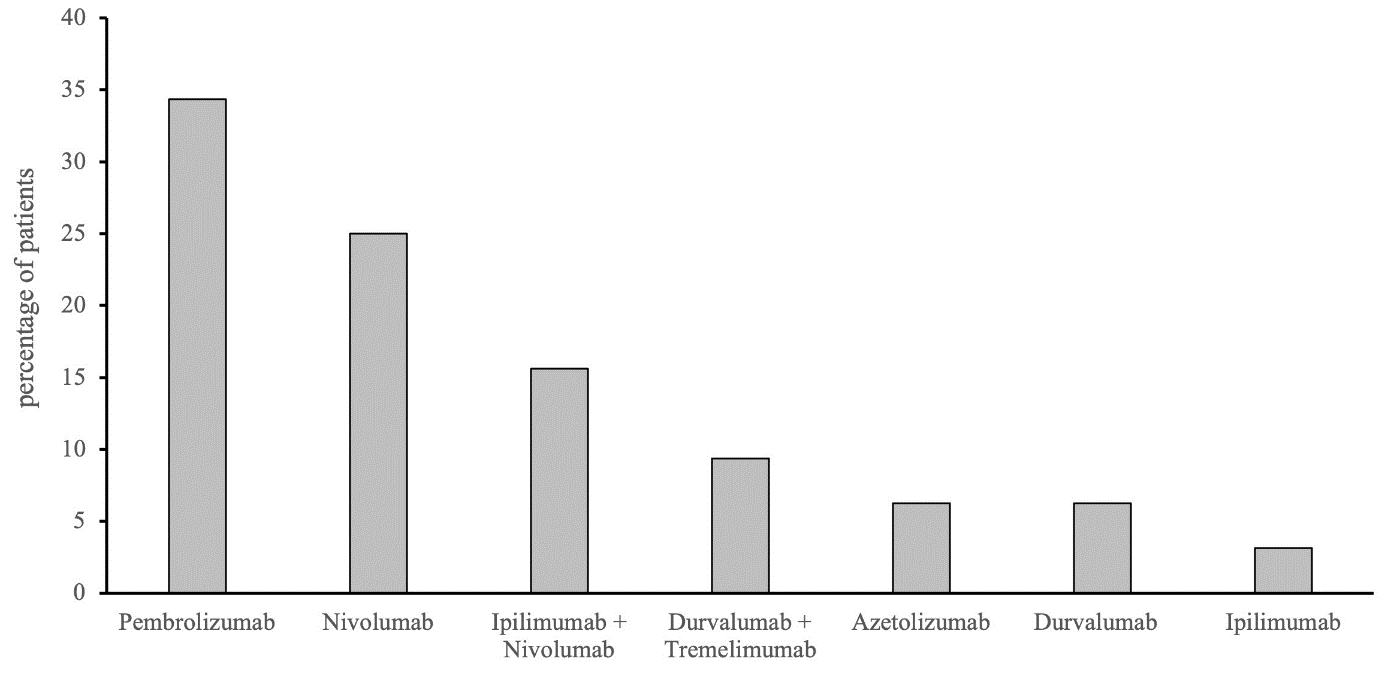

Supplement: Supplementary file 6 — Supplementary Material 6 [file 40644_2023_580_MOESM6_ESM.docx]
