# Supplementary material for: Patterns of pseudoprogression across different cancer entities treated with immune checkpoint inhibitors
Source: Cancer Imaging. 2023 Jun 8;23:58. doi: 10.1186/s40644-023-00580-9 (PMC10249323; doi:10.1186/s40644-023-00580-9)
Supplement: Supplementary file 7 — Supplementary Material 7 [file 40644_2023_580_MOESM7_ESM.docx]

**Table S4. Comparison of patients according to lung tumor versus other tumor**

|  | Lung tumor  (N = 7) | Other tumor  (N = 25) | P value |
| --- | --- | --- | --- |
| **PsPD at FU1** | 86.6 % (N = 6) | 80.0 % (N = 20) | 0.731 |
| **Max. increase of TL (cm)** | 22.4 ± 35.7 | 10.2 ± 17.2 | 0.210 |
| **Max. decrease of TL (cm)** | -20.0 ± 23.5 | -16.7 ± 14.0 | 0.653 |
| **Presence of irAE** | 42.9 % (N = 3) | 45.8 % (N = 11) | 0.889 |
| **Elevated LDH** | 14.3 % (N = 1) | 19.0 % (N = 4) | 0.776 |
| **Concordant tumor markers** | 0.0 % (N = 0) | 8.0 % (N = 2) | 0.023 |

PsPD pseudoprogression, irAE immune-related adverse event, TL target lesion sum, max. maximum, LDH lactate dehydrogenase, FU follow-up examination
