# Supplementary material for: Patterns of pseudoprogression across different cancer entities treated with immune checkpoint inhibitors
Source: Cancer Imaging. 2023 Jun 8;23:58. doi: 10.1186/s40644-023-00580-9 (PMC10249323; doi:10.1186/s40644-023-00580-9)
Supplement: Supplementary file 8 — Supplementary Material 8 [file 40644_2023_580_MOESM8_ESM.docx]

**Table S5. Comparison of patients according to Pembrolizumab versus other ICI**

|  | Pembrolizumab monotherapy  (N = 11) | Other ICI  (N = 21) | P value |
| --- | --- | --- | --- |
| **PsPD at FU1** | 81.8 % (N = 9) | 80.1 % (N = 17) | 0.952 |
| **Max. increase of TL (cm)** | 11.8 ± 33.1 | 13.4 ± 15.2 | 0.846 |
| **Max. decrease of TL (cm)** | -20.8 ± 20.7 | -15.7 ± 13.9 | 0.444 |
| **Presence of irAE** | 27.3 % (N = 3) | 52.4 % (N = 11) | 0.242 |
| **Elevated LDH** | 18.2 % (N = 2) | 17.6 % (N = 3) | 0.971 |
| **Concordant tumor markers** | 9.1 % (N = 1) | 4.7 % (N = 1) | 0.862 |

PsPD pseudoprogression, irAE immune-related adverse event, TL target lesion sum, max. maximum, LDH lactate dehydrogenase, FU follow-up examination, ICI immune checkpoint inhibitor therapy
