# Supplementary material for: Patterns of pseudoprogression across different cancer entities treated with immune checkpoint inhibitors
Source: Cancer Imaging. 2023 Jun 8;23:58. doi: 10.1186/s40644-023-00580-9 (PMC10249323; doi:10.1186/s40644-023-00580-9)
Supplement: Supplementary file 9 — Supplementary Material 9 [file 40644_2023_580_MOESM9_ESM.docx]

**Table S6. Comparison of patients according to Nivolumab versus other ICI**

|  | Nivolumab monotherapy  (N = 7) | Other ICI  (N = 25) | P value |
| --- | --- | --- | --- |
| **PsPD at FU1** | 71.4 % (N = 5) | 84.0 % (N = 21) | 0.451 |
| **Max. increase of TL (cm)** | 13.9 ± 13.3 | 12.6 ± 24.6 | 0.890 |
| **Max. decrease of TL (cm)** | -21.1 ± 18.5 | -16.4 ± 15.9 | 0.516 |
| **Presence of irAE** | 57.1 % (N = 4) | 41.7 % (N = 10) | 0.469 |
| **Elevated LDH** | 20.0 % (N = 1) | 8.0 % (N = 2) | 0.890 |
| **Concordant tumor markers** | 0.0 % (N = 0) | 12.5 % (N = 1) | 0.512 |

PsPD pseudoprogression, irAE immune-related adverse event, TL target lesion sum, max. maximum, LDH lactate dehydrogenase, FU follow-up examination, ICI immune checkpoint inhibitor therapy
