# Supplementary material for: Patterns of pseudoprogression across different cancer entities treated with immune checkpoint inhibitors
Source: Cancer Imaging. 2023 Jun 8;23:58. doi: 10.1186/s40644-023-00580-9 (PMC10249323; doi:10.1186/s40644-023-00580-9)
Supplement: Supplementary file 10 — Supplementary Material 10 [file 40644_2023_580_MOESM10_ESM.docx]

**Table S7. Comparison of patients according to ICI monotherapy versus association of ICI**

|  | ICI monotherapy  (N = 24) | Association of  ICI  (N = 8) | P value |
| --- | --- | --- | --- |
| **PsPD at FU1** | 70.2 % (N = 19) | 87.5 % (N = 7) | 0.601 |
| **Max. increase of TL (cm)** | 11.9 ± 24.1 | 16.0 ± 17.5 | 0.657 |
| **Max. decrease of TL (cm)** | -19.1 ± 17.7 | -11.5 ± 8,6 | 0.322 |
| **Presence of irAE** | 29.1 % (N = 9) | 62.5 % (N = 5) | 0.253 |
| **Elevated LDH** | 14.3 % (N = 3) | 28.6 % (N = 2) | 0.393 |
| **Concordant tumor markers** | 4.1 % (N = 1) | 12.5 % (N = 1) | 0.476 |

PsPD pseudoprogression, irAE immune-related adverse event, TL target lesion sum, max. maximum, LDH lactate dehydrogenase, FU follow-up examination, ICI immune checkpoint inhibitor therapy
